# Dataset of short-term prediction of CO_2_ concentration based on a wireless sensor network

**DOI:** 10.1016/j.dib.2020.105924

**Published:** 2020-06-25

**Authors:** Ari Wibisono, Hanif Arief Wisesa, Novian Habibie, Aulia Arshad, Aditya Murdha, Wisnu Jatmiko, Ahmad Gamal, Indra Hermawan, Siti Aminah

**Affiliations:** a Faculty of Computer Science, Universitas Indonesia, Kampus UI Depok, Indonesia; b University of Freiburg, Fahnenbergplatz, 79085 Freiburg im Breisgau, Germany; c Faculty of Engineering, Universitas Indonesia, Kampus UI Depok, Indonesia

**Keywords:** CO_2_ prediction, CO_2_ monitoring system, Wireless sensor network, Prediction system, IoT system

## Abstract

This CO_2_ data is gathered from WSN (Wireless Sensor Network) sensors that is placed in some areas. To make this observation framework run effectively, examining the relationships between factors is required. We can utilize multiple wireless sensor devices. There are three parts of the system, including the sensor device, the sink node device, and the server. We use those devices to acquire data over a three-month period. In terms of the server infrastructure, we utilized an application server, a user interface server, and a database server to store our data. This study built a WSN framework for CO_2_ observations. We investigate, analyze, and predict the level of CO_2_, and the results have been collected. The Random Forest algorithm achieved a 0.82 R2 Score.

Specification table

**Table:** 

Subject	Computer Science Applications
Specific Subject Area	Computer Science Applications, CO_2_ Prediction
Type of Data	Table Image Chart Graph Figure
How data were acquired	Data is acquired from three sensors. Those sensors are built by authors for the process of data collection. These sensors operated in 24 h and send the data to servers for every second. We have acquired the CO_2_ data for three months period.
Data format	Raw (.csv) comma separated file
Parameters for data collection	The data analysis process is done after the data have been collected over three months. The data acquired for 24 h. There are 6 million instances of sensor readings for the three months. After that, the data are cleared, engineering features are carried out, and models are made to make predictions.
Description of data collection	The data retrieval process is done by placing three nodes in an area. Each node will be connected to 1 other node to be able to send the data from the sensor readings. There is one node sink that is equipped with a GSM to be able to send data to servers that have been online. We have built the hardware and software for the data acquisition process. Data field: 1. Record id - Increment record id (unique) 2. Linux timestamp- convert from linux timestamp to time format https://www.epochconverter.com/ 3. CO_2_ concentration - in ppm (part per million) 4. Temperature - Temperature in °C 5. Humidity - (Humidity in percentage%) 6. Light intensity - Light Intensity, approx. 0 – 1000 7. Node ID Node 0, Node 1, and Node 9
	The data generated from the sensors can be used to form a model for measuring and predicting future CO_2_ measurements. Because the time interval for recording CO_2_ level is very short, many data are generated. This makes it very possible to predict CO_2_ values based on time. This method can be very feasibly implemented in various places The data generated are approximately 6 million instances. The prediction is done by dividing the data into 2 parts, namely, 80% of the data are used for training the machine learning models and 20% of the data are used for testing. We do 10-fold cross-validation to measure the performance of each algorithm against the sensor acquisition data. The metrics used are standard metrics, namely, the Mean Absolute Error (MAE), the Root Mean Square Error (RMSE), and the R2 Score. Fig. 1, Fig. 5, Fig. 6, Fig. 7 are our framework design to gathered environment data. Fig. 2 shows the frequency distribution of each feature. In Fig. 2. (a) is the graph for the CO2 data. The most frequent value for CO2 data is approximately 200 - 300 ppm. As we know, the global CO2 concentration is approximately 450 ppm. The measurement results show that the CO2 concentration is are lower than the global condition. Fig. 2. (b) shows the results of the air temperature measurements. In general, the most frequent value is between 25 and 30 °Celsius. Sometimes the temperature can drop to below 20° when the weather is cold and increase to more than 35° during the day. These data are taken in a tropical environment, which means that there is no winter, summer, autumn, or spring condition.
	Based on the observations in Table 1, there are two directions, positive and negative correlations. These are the conclusions of the correlation analysis of the data. Regarding the correlation of CO_2_ - Air Humidity, from the analysis of the gathered data, the correlation between CO_2_ and humidity is consistently more than 0.6. The mean correlation is 0.63. A strong correlation with a positive direction is picturized by the Cohen Scale. This means that the two parameters move in the same direction. According to Fiddbruk, the correlation between CO_2_ and humidity happens in indoor situations since humans exhale CO_2_, which produces water vapor [7]. This may also be true for the outdoor situation since there are many organisms and machinery that emit CO_2_. As can be seen in Fig. 8, the MAE measurement results show that a fairly low error is obtained by the Random Forest (RF) algorithm with an average MAE of 37.07 and that for the Gradient Boosting Regressor (GBR) was 36.77. High errors are obtained by the Linear Regression (LR) and Ridge Regression (RR) at 63.53. The Decision Tree Regressor (DT) has a fairly small error of 45.3. be seen in Fig. 9, the RMSE measurement results show that a fairly low error is obtained by the Random Forest (RF) algorithm with an average RMSE of 51.2 and that for the gradient Boosting Regressor (GBR) was 49.06. High errors are obtained by the Linear Regression (LR) and Ridge Regression (RR) at 91.9. The Decision Tree Regressor (DT) has a fairly small error of 63.32. To assess the quality of the prediction results, we evaluate the R2 score, which is in the range of 0 - 1. The measurement results of the 10-fold cross validation are described in the form of boxplots, where one boxplot can represent a general measurement result, the highest measurement results, the lowest measurement results, and the median. As can be seen in Fig. 10, the measurement results of the R2 Score show that a high enough score is obtained by the Random Forest (RF) algorithm with an average R2 Score of 0.78 and that for the Gradient Boosting Regressor (GBR) was 0.82. Low R2 Score results are obtained by the Linear Regression (LR) and Ridge Regression (RR) at 0.43. The Decree Tree Regressor (DT) has a fairly large R2 of 0.72. All of the dataset are available in Mendeley data repository.
Data source location	Institution: Faculty of Computer Science Universitas Indonesia City/Town/Region: Depok/West Java Country:Indonesia
Data accessibility	Upload to mendeley https://data.mendeley.com/datasets/6d798dkhpz/draft?a=502e7fa1-e1b4-4931-99eb-1aeb4eec4f13 Source code: https://github.com/WSN-1231/.
Related research article	Habibie et al., “Comparative study of lightweight secure multiroute communication system in low cost wireless sensor network for CO_2_ monitoring,” 2016 International Workshop on Big Data and Information Security (IWBIS), Jakarta, 2016, pp. 145–150, doi: 10.1109/IWBIS.2016.7872904. A. Arshad, N. Habibie, A. Wibisono, P. Mursanto, W. S. Nugroho and W. Jatmiko, “Sensor node for data sampling and correlation analysis of CO_2_ concentration with air humidity, temperature, and light intensity,” 2016 International Conference on Advanced Computer Science and Information Systems (ICACSIS), Malang, 2016, pp. 111–116, doi: 10.1109/ICACSIS.2016.7872793.

## Value of data

• This data consists of the results of environmental sensor readings which useful to support monitoring and prediction of environmental changes. This data is important because it gathered from several sensors regarding environmental conditions. The parameters measured are temperature, CO_2_ concentration, humidity, and light intensity.
• All institutions, governments, and private sectors that have intention on monitoring and predicting environmental conditions will benefit from this data.
• The added value of this data is the fast time-taking interval of the data gathering process. The sensors gathered the environment condition every 1 s for 24 h.
• A framework to gathered the environment data has been proposed in this data. It is implemented using WSN to observe CO_2_ concentration, temperature, light intensity, and humidity. Framework integration between hardware devices, protocols, wireless sensor networks, and servers.
• This data is a representation for evaluating and predicting rapid environmental changes within a certain period. Data Analysis correlation factors that influence the measurement of CO_2_ concentration
• In short-term use, this data will be useful for recognizing the rapidly changing environmental conditions of an area. In the long term, this data can recognize local changes that will shape the behavior of environmental conditions over a long period.

## 1 Data

The data retrieval process is done by placing three nodes in an area. Each node will be connected to 1 other node to be able to send the data from the sensor readings. There is one node sink that is equipped with a GSM to be able to send data to servers that have been online. Each sensor is equipped with a 12 V 65A battery. The battery's ability to supply power to the equipment is five days. Every five days, the battery is replaced for each node.

The most feasible method to gather the data and monitor the data is by using a Wireless Sensor Network (WSN). The main function of a WSN is to monitor several remote [1,2]. A WSN provides a framework using a network of sensors to observe those areas. It can primarily be used to monitor natural resources, telecommunications, and others. There has been much research that was conducted related to Wireless Sensor Networks (WSNs). Oiha et al. used a WSN to monitor farming [2] and Rashid et al. used the framework for urban territory monitoring. Mekki et al. used a WSN to monitor the CO_2_, temperature, and dampness light to observe the natural environment [3]. Another research conducted by Sudarrono examined secure data transmission using a WSN by using light encryption that is implemented inside the WSN framework [4], [5], [6].

The main advantage of using the WSN framework is its size and cost. The sensors in this framework are very compact and affordable. Additionally, it is very efficient in terms of its power consumption. This makes it more feasible for it to be implemented in remote areas where remote monitoring will be done using microcontrollers. However, its computational and memory power are limited due to the low-end grade of the controller. Therefore, it is a challenge to implement many available algorithms due to the limitation of this computational resource. A correct and suitable algorithm needs to be implemented in the WSN.

### 1.1 Data acquisition process

Throughout the span of three months, we obtained 6 million instances to be used to analyze and model CO_2_ behavior. The Data Logging and sensor nodes’ data acquisition occur every 1 s.

**Fig. 1:**
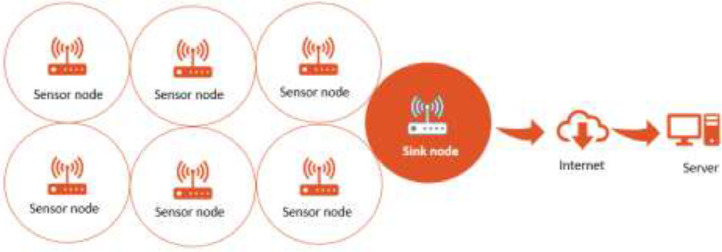
Design of the WSN architecture.

**Fig. 2:**
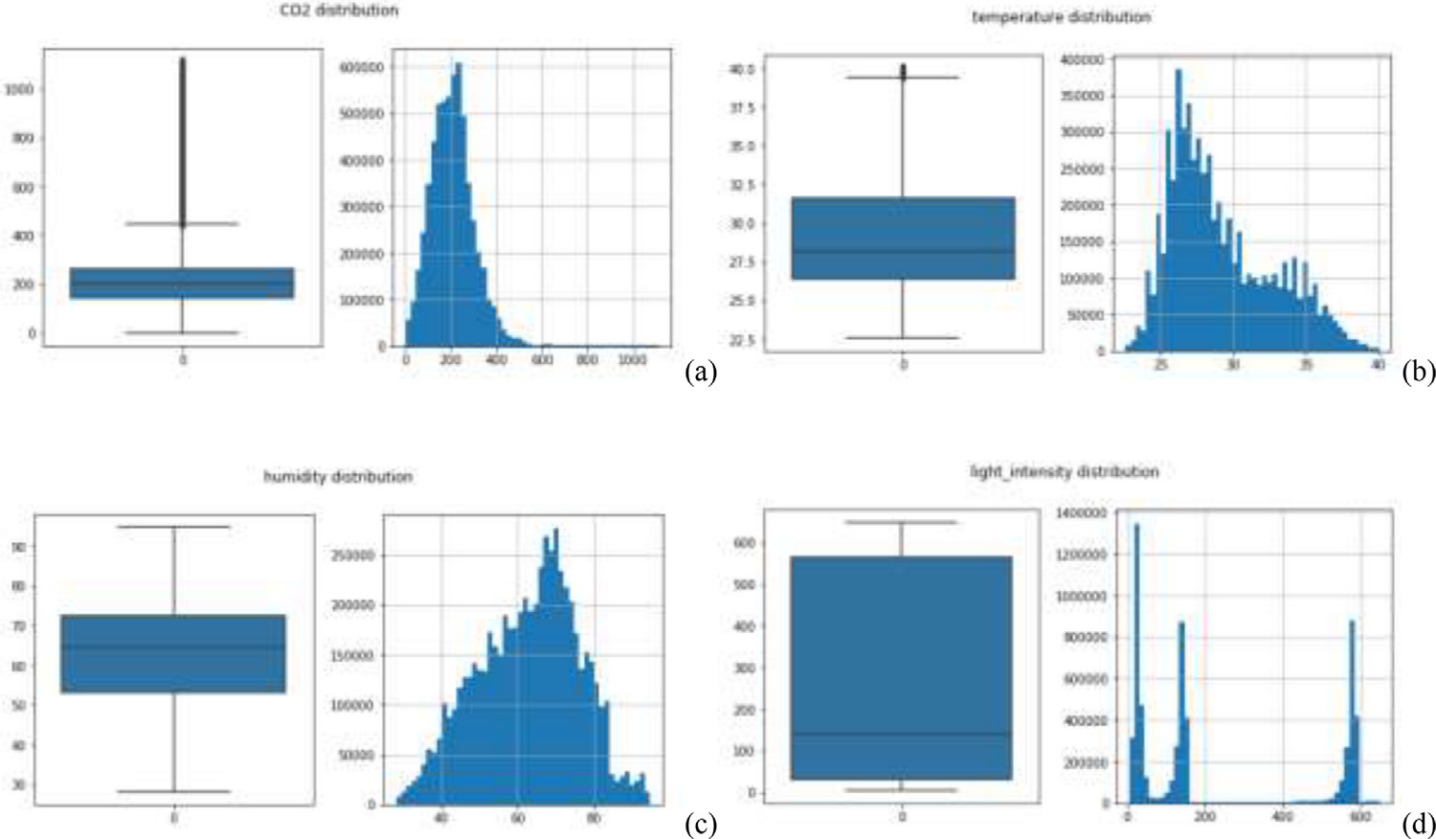
Distribution frequencies of the data features: (a) CO_2_, (b) temperature, (c) humidity, and (d) light intensity.

Fig. 1 shows the frequency distribution of each feature. In Fig. 2(a) is the graph for the CO_2_ data. The most frequent value for CO_2_ data is approximately 200 - 300 ppm. As we know, the global CO_2_ concentration is approximately 450 ppm. The measurement results show that the CO_2_ concentration is are lower than the global condition. Fig. 2(b) shows the results of the air temperature measurements. In general, the most frequent value is between 25 and 30 °Celsius. Sometimes the temperature can drop to below 20° when the weather is cold and increase to more than 35° during the day. These data are taken in a tropical environment, which means that there is no winter, summer, autumn, or spring condition.

Fig. 2(c) shows the frequency distribution for the air humidity data. The most frequent value is between 60 - 70%. As we know, the tropics usually have a high level of humidity. Fig. 2(d) shows the frequency distribution of the light intensity. In the frequency distribution in Fig. 2(d), there are two significant peak frequencies, among others. First is the intensity of the light between 0 and 200, which is the change in the intensity from the evening - night and morning to noon. The next frequency distribution is between 500 - 600, which is the light intensity during the day.

### 1.2 Data analysis

The data analysis process is done after the data have been collected over three months. There are 6 million instances of sensor readings for the three months. After that, the data are cleared, engineering features are carried out, and models are made to make predictions.

**Fig. 3:**
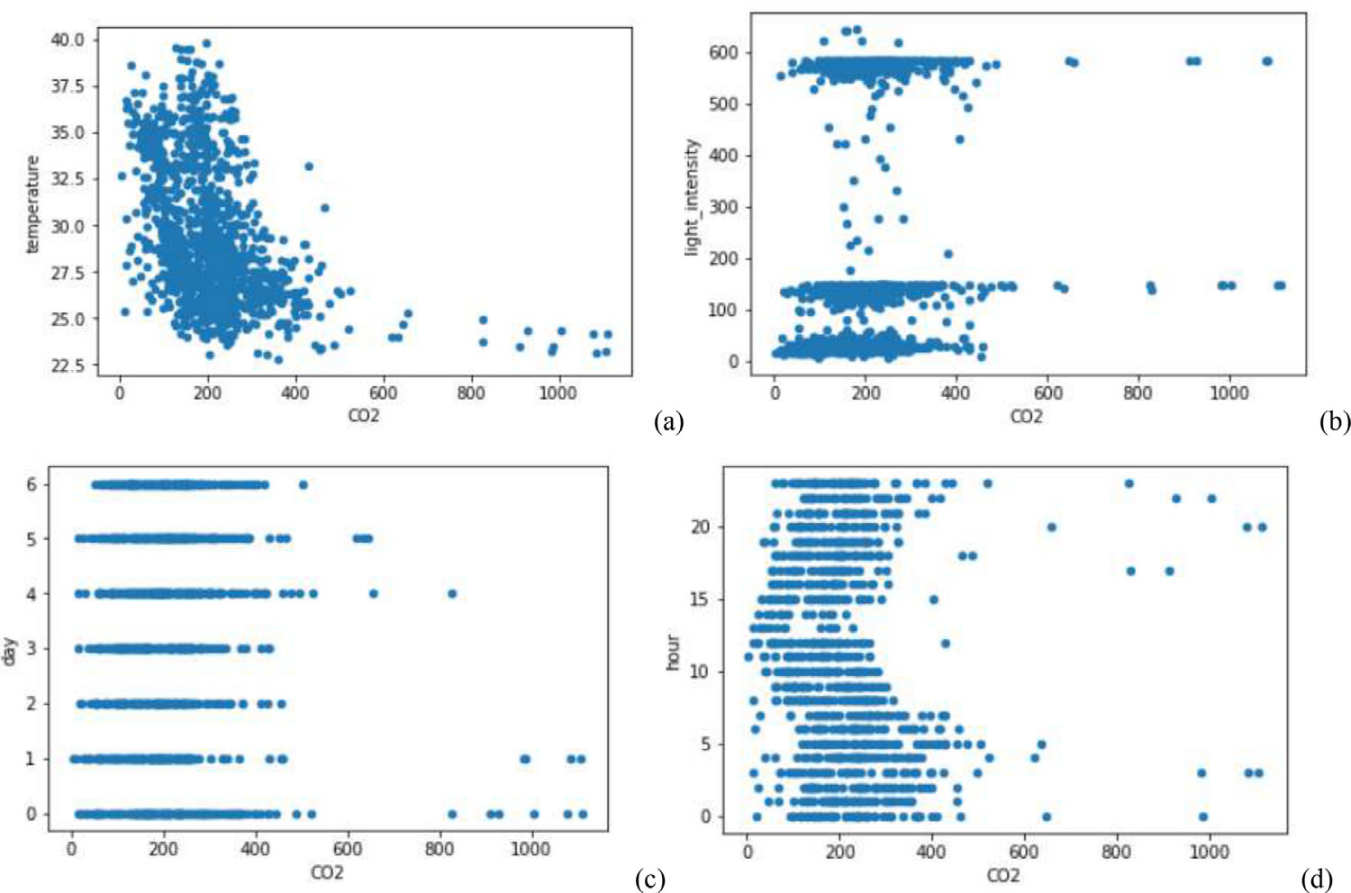
Correlation of (a) Temperature, (b) Light Intensity, (c) day, and (d) hour with CO_2_.

**Fig. 4:**
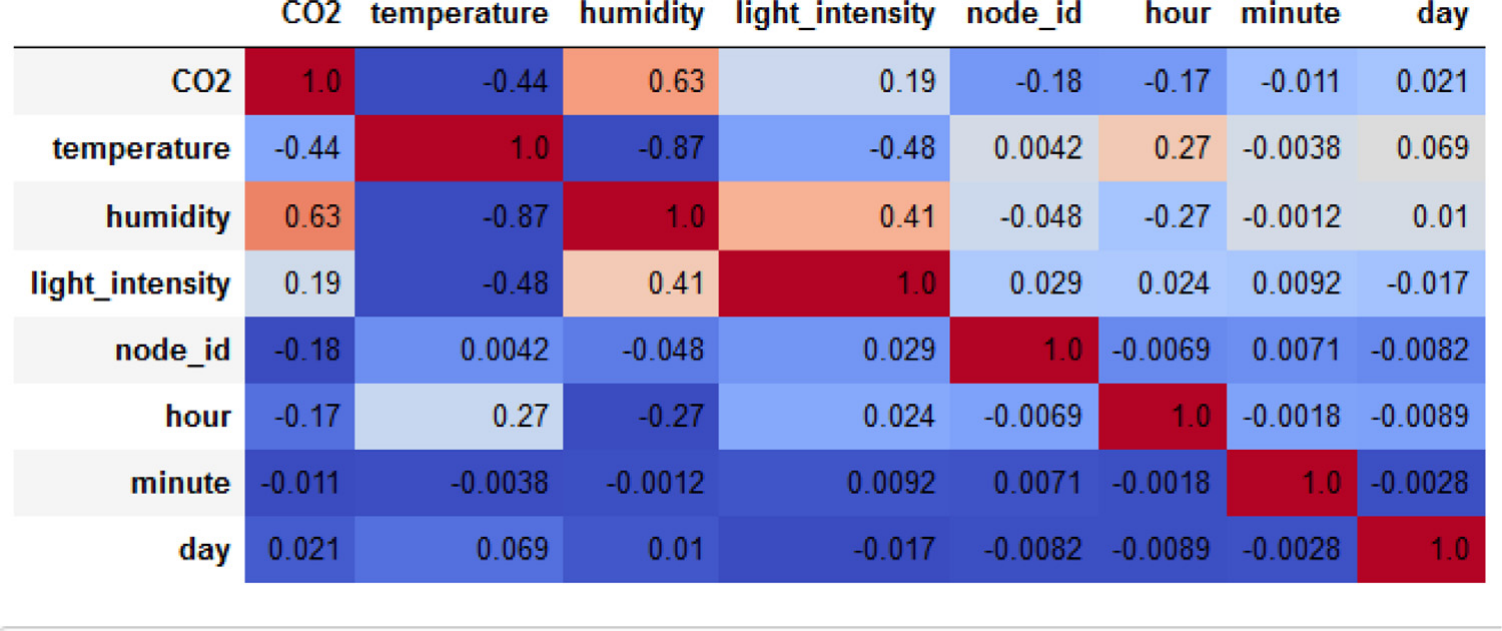
Pearson correlation scores of features.

Fig. 3 shows a description of the correlation of each feature with the CO2 variable. It can be seen in Fig. 3(a) that there is a decreasing trend between the temperature and CO2 features. This shows a strong negative correlation between the two features. From the results of the measurement of the correlation score between the two variables in Fig. 4, the two variables have a high correlation score of 0.63. In Fig. 3(b), (c) and (d), there is no visible up or downtrend with respect to the CO_2_ variable such that the correlations given in Fig. 8 is not too large between these variables with respect to CO2, which are below 0.2.

The correlation analysis was conducted using the 24 h measurements. Each day may have different data characteristics. Therefore, we change the analysis to daily analysis. An example of the reason for this is that the CO_2_ concentration during working days is relatively higher than the concentration during the weekend.

Based on the observations in Table 1, there are two directions, positive and negative correlations. These are the conclusions of the correlation analysis of the data. Regarding the correlation of CO_2_ - Air Humidity, from the analysis of the gathered data, the correlation between CO_2_ and humidity is consistently more than 0.6. The mean correlation is 0.63. A strong correlation with a positive direction is picturized by the Cohen Scale. This means that the two parameters move in the same direction. According to Fiddbruk, the correlation between CO_2_ and humidity happens in indoor situations since humans exhale CO_2_, which produces water vapor [7]. This may also be true for the outdoor situation since there are many organisms and machinery that emit CO_2_.

**Table 1:** Correlation between light power, air moistness, and air temperature with CO_2_.

Parameters	Correlation value	Result
CO_2_ + Humidity	0.63	+ Strong correlation (A)
CO_2_ + Temperature	- 0.44	- normal Correlation (B)
CO_2_ + Light Intensity	0.19	+ weak correlation (C)
CO_2_ + Hour	−0.17	- weak correlation (C)
CO_2_ + Minute	−0.011	- weak correlation (D)
CO_2_ + Day	0.021	+ weak correlation (D)

One of the researchers concludes that the air temperature is not correlated with the CO_2_ concentration, where the CO_2_ does not contribute to temperature changes [8]. However, in this dataset, regarding the correlation of CO_2_ - Air Temperature, there is a consistent correlation of CO_2_ and temperature. It resulted in a Pearson Correlation Score of −0.44. Based on our analysis, the correlation between the light intensity and CO_2_ concentration is weak. It is also negative. From the data samples, almost all of them have weak correlations. This condition occurs due to the effect of photosynthesis in plants, where the higher light intensity will reduce the CO_2_ intensity surrounding the plants (since the photosynthesis process uses CO_2_) [9,10]. However, the results may vary in open air since the original work was done in a closed room.

The time parameter is also considered in this analysis. The results were −0.17, −0.011, and 0.021 for CO_2_ + Hour, CO_2_ + Minute, and CO_2_ + Day, respectively. Although the correlation score of the time parameter is below 0,2, we still need to consider these parameters as features for the machine learning evaluation since these time parameters influence the temperature and light intensity. The temperature, light intensity, and humidity are influenced by these time parameters.

## 2 Experimental, system design, and prediction methods

Carbon dioxide (CO_2_) is a gas that exists in our atmosphere. In the correct amount, it could balance the atmosphere and create a more stable condition. In recent years, the concentration of CO_2_ around the world has increased very rapidly with an increase of 21.12 ppm from 2006 to 2015 [11]. The vast increase in the CO_2_ concentration in our atmosphere could cause an imbalanced air composition, which could lead to several chronic diseases. These diseases include respiratory issues, vision issues, and other complications [12]. Additionally, the rise in the CO_2_ concentration could also cause an increase in the global temperature. The agricultural sector could also be affected by the increase in the CO_2_ concentration since an excessive amount of CO_2_ being processed during the photosynthesis of plants could actually reduce the nutrients contained in the crops [13,14].

To tackle this issue, more detailed monitoring of the CO_2_ concentration is required. The data gathered from the monitoring process will be used for decision making in order to hamper the increase in the CO_2_ concentration. To do this, we need to have a state-of-the-art CO_2_ monitoring system. A feasible solution is using a Wireless Sensor Network (WSN) to acquire these CO_2_ concentration data. The sensors are placed in certain locations to gather data, which are then shared with the other sensors in the network to analyze the CO_2_ data.

To assist the data analysis process, other additional parameters are required so that the exact state is known in detail. These parameters include the temperature, air humidity, and light intensity. However, not all of these parameters will contribute substantially to the analytical process. Therefore, we need to select the parameters that will help the CO_2_ data analysis process. To do this, a correlation evaluation between the parameters and the CO_2_ concentration is needed.

Various research has been done in order to examine the correlation of those parameters with the CO_2_ concentration. Li, et al. analyzed the concentration of CO_2_ in different altitudes in the atmosphere [15]. To do this, the authors attached a CO_2_ sensor on an air balloon. The results showed that the CO_2_ concentration is lower at the higher altitudes of our atmosphere. This means that the altitude is inversely correlated with the CO_2_ concentration. Another study in 2013 analyzed the correlation of the CO_2_ concentration with another parameter. Katarzyna et al. analyzed the correlation between the indoor CO_2_ concentration and air humidity [7]. Lazovic et al. also studied the correlation of the humidity and air temperature [16]. Another correlation study conducted by Soares analyzed the correlation between CO_2_ and the temperature in order to analyze global climate change. A. Arshad, et al. conducted data sampling and a correlation test for CO_2_ [17].

### 2.1 System design

#### 2.1.1 Hardware design

There are a few parts in our hardware such as the timer RTC module, the Radio Frequency (RF) communication module sensor, the light intensity sensor, the integrated environmental sensor (CO_2_ sensor, temperature sensor, and humidity sensor), and the 256 KB Microcontroller. We utilize a Single Board Computer (SBC) for temporary storage. The microcontroller controls the various sensors that transmit the captured data and stores the captured data in a Raspberry Pi 2 (Single Board Computer). After the data from many nodes are stored in our SBC, we send all captured data to our server using a 4 G connection via the internet.

#### 2.1.2 Software design

**Fig. 5:**
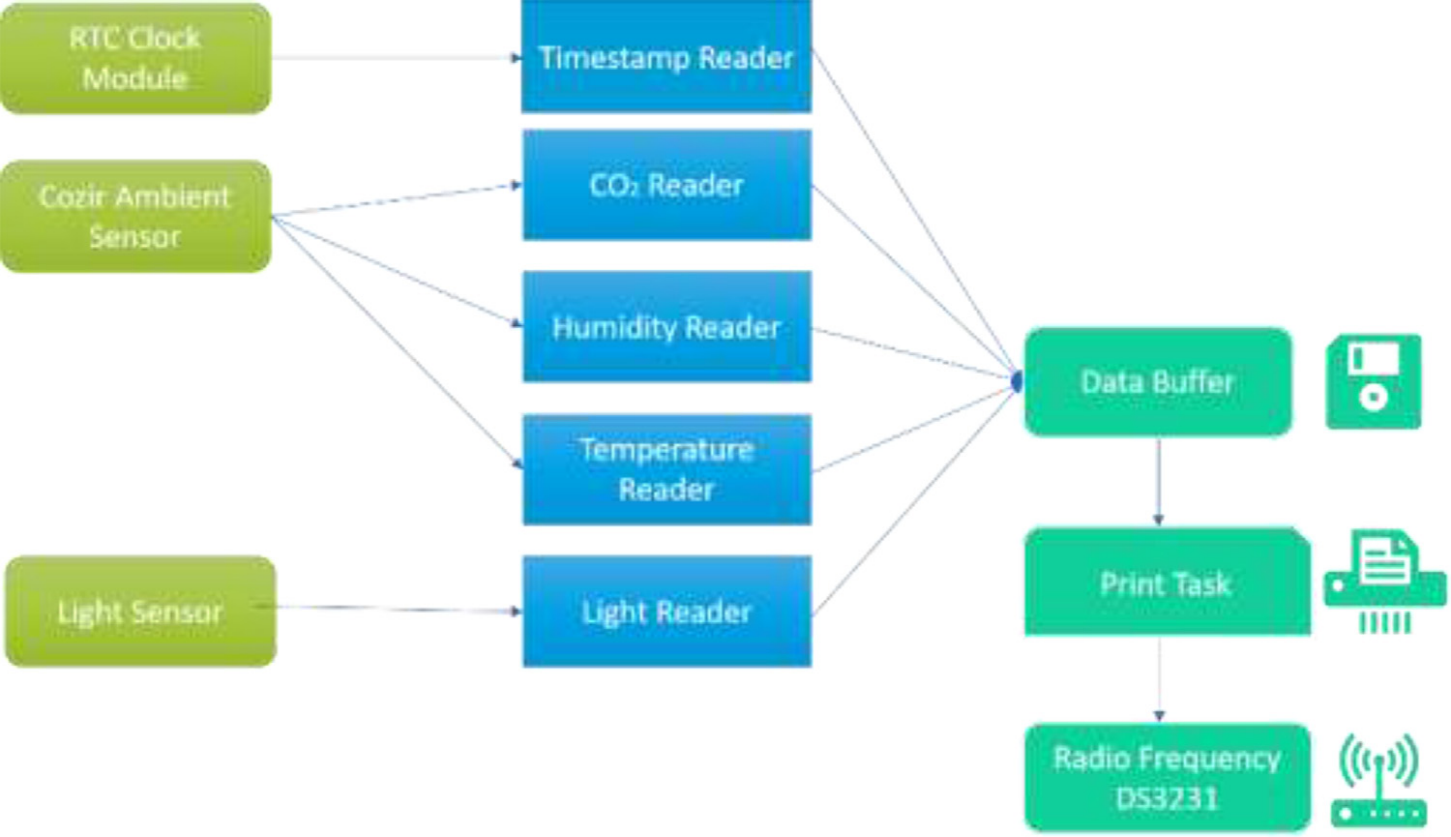
RTOS configuration.

The Raspberry Pi 2 has a Linux Raspbian Operating System. Qt5 is used to develop the temporary storage, which is compiled in the Raspberry Pi 2. The temporary storage is connected to the Microcontroller. The 256 KB Microcontroller uses the Real Time Operating System (RTOS). The RTOS can perform multitasking, which is divided into tasks independent of each other, and is also better than the primitive interrupt for an embedded system [18]. The process inside the sensor node is divided into five tasks: the retrieval of CO_2_, temperature, humidity, and light intensity data and integration. The RTOS that is used in this study is FreeRTOS [19]. The configuration can be found in Fig. 5.

#### 2.1.3 WSN design

**Fig. 6:**
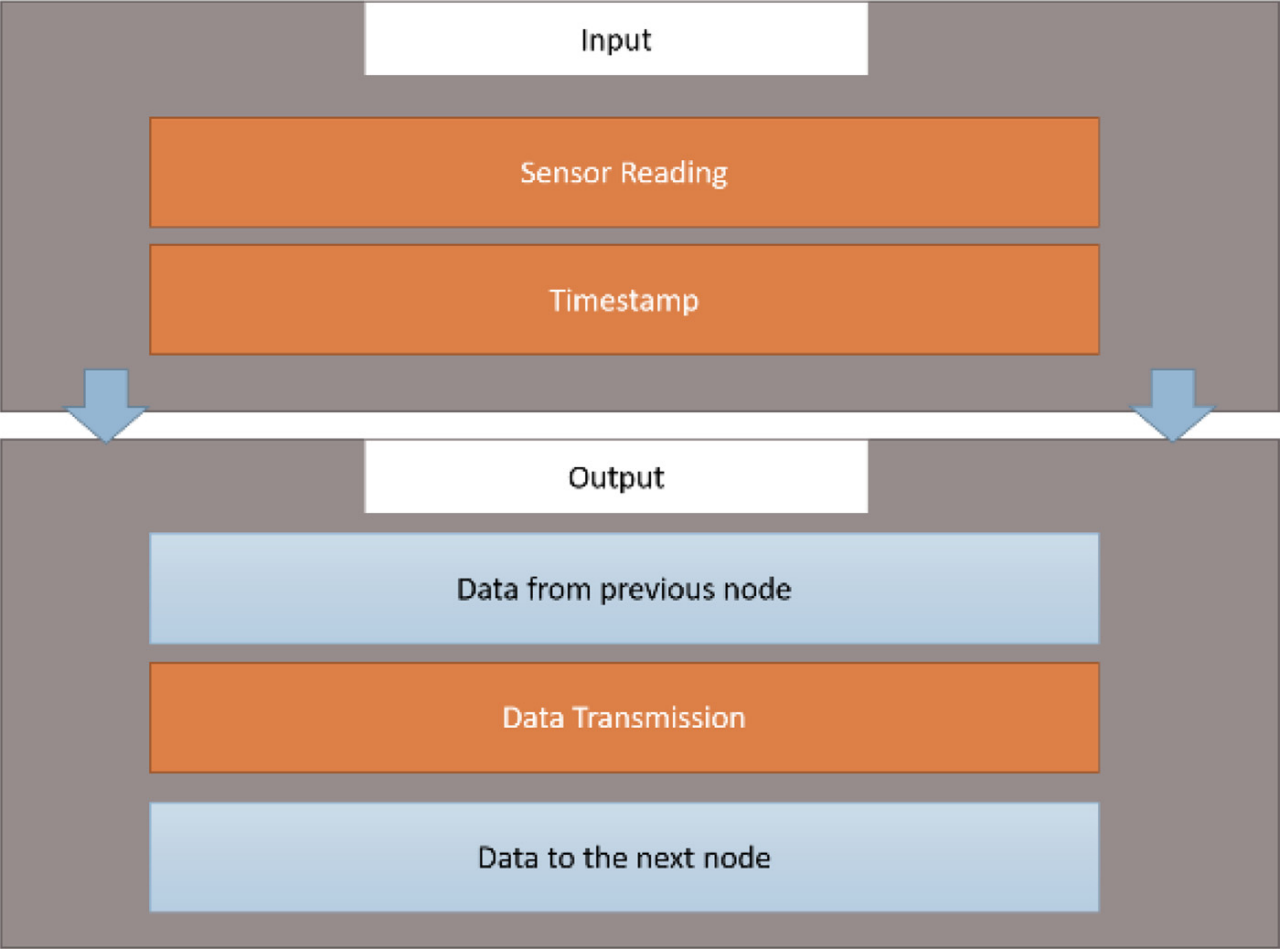
The data transmission flow.

In this research, the WSN system will also be integrated with the server. As mentioned in the previous section, the input data will be gathered by the sensor nodes, which are installed according to the testing. The data that were gathered by these nodes will be stored inside the centralized storage. The temporary storage will act as the producer that transmits the data to the server web service. The server is the consumer, which will gather the data and relay it to the SQL database. Fig. 6. shows the information flow in the sensor node.

**Fig. 7:**
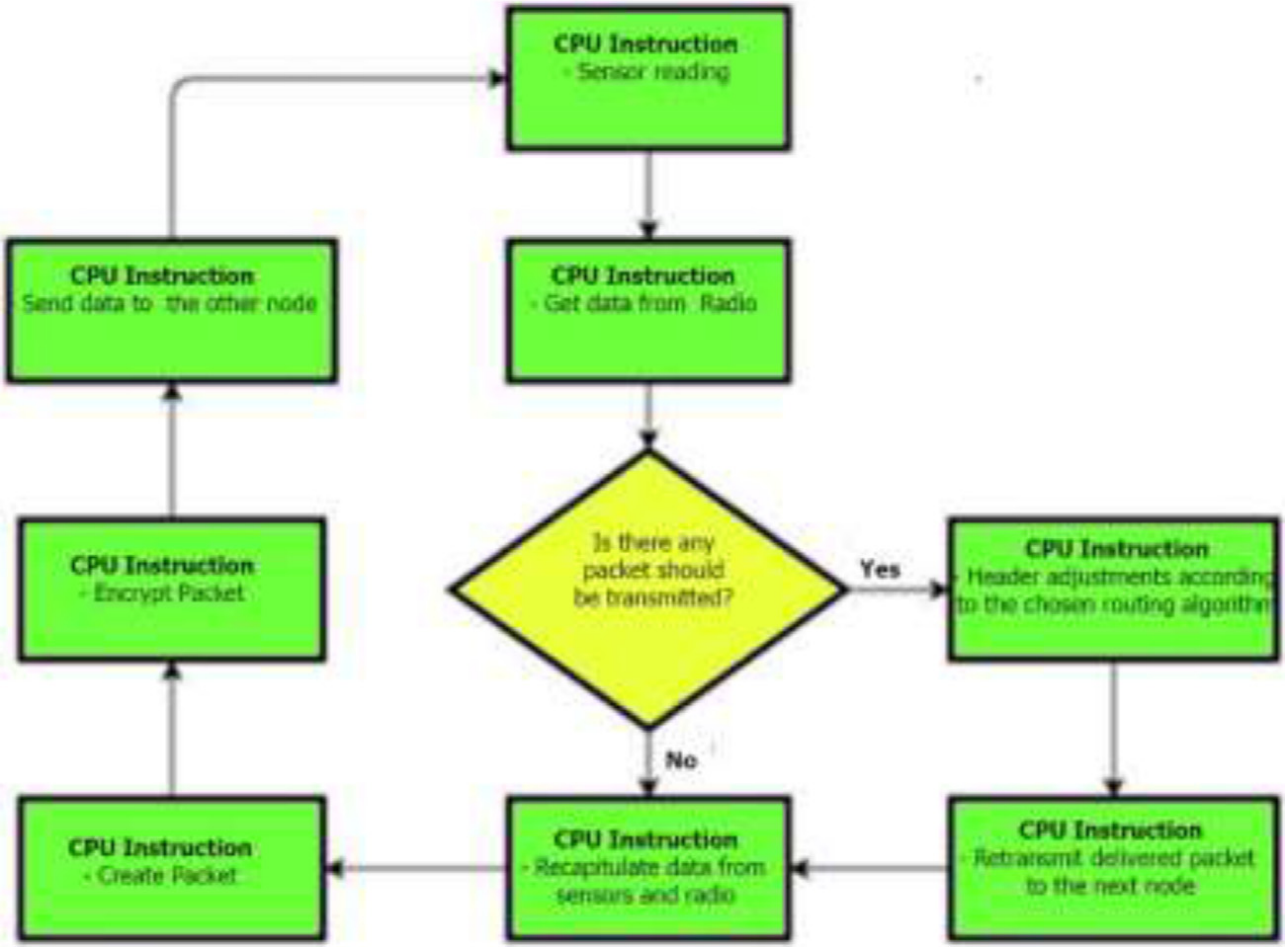
RTOS tasks in the microcontroller.

Multiple nodes will establish a radio-based communication network in the zone. There are two types of WSN nodes: the source node and the sink node. The source node acquires data from the sensor, and the sink node is responsible for transmitting the data acquired by the source node to the server. These two processes should be done simultaneously during their execution. Sensor tasks are implemented in the Microcontroller by using the RTOS. The details of the RTOS task flow in the Microcontroller are described in Fig. 7.

### 2.2 Experiment result and prediction system

This experiment obtains time series data to analyze the correlation of one parameter with the other parameters. The sampling has been done using the proposed WSN nodes in a specific area.

In the experiment, the sensor nodes were placed on a road with 1.5 m between the nodes and the road. Data logging was conducted over two weeks. According to Li, the CO_2_ sampling that is conducted at a very low height will result in a higher concentration of CO_2_ [15]. Therefore, we have placed the nodes as low as possible, which is 1 m due to safety reasons.

The data generated from the sensors can be used to form a model for measuring and predicting future CO_2_ measurements. Because the time interval for recording CO_2_ level is very short, many data are generated. This makes it very possible to predict CO_2_ values based on time. This method can be very feasibly implemented in various places.

The data generated are approximately 6 million instances. The prediction is done by dividing the data into 2 parts, namely, 80% of the data are used for training the machine learning models and 20% of the data are used for testing. We do 10-fold cross-validation to measure the performance of each algorithm against the sensor acquisition data. The metrics used are standard metrics, namely, the Mean Absolute Error (MAE), the Root Mean Square Error (RMSE), and the R2 Score.

**Fig. 8:**
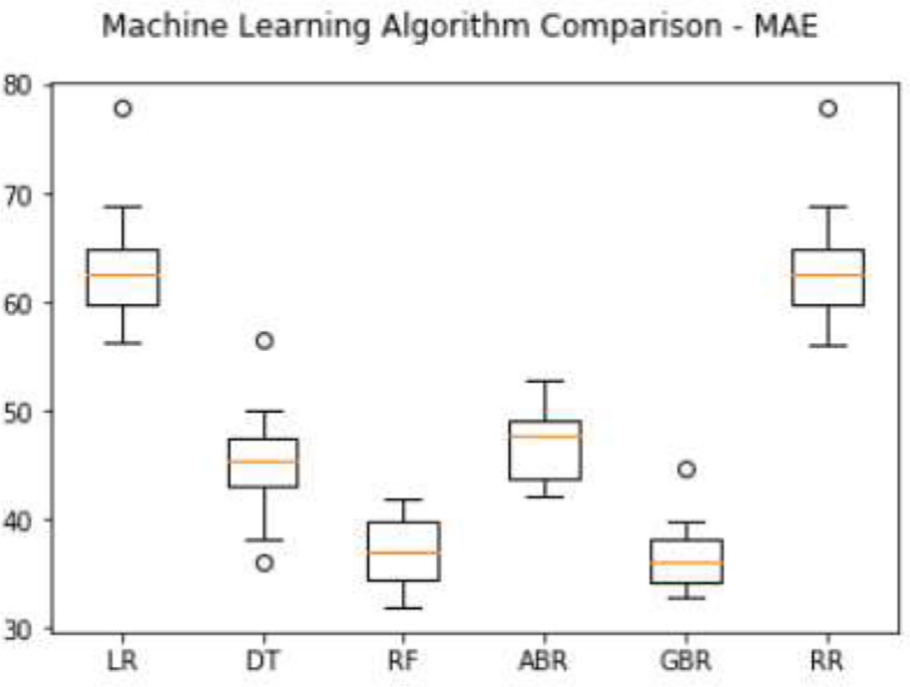
MAE comparison result.

The measurement results of the 10-fold cross validation are described in the form of boxplots, where one boxplot can represent a general measurement result, the highest measurement results, the lowest measurement results, and the median. As can be seen in Fig. 8, the MAE measurement results show that a fairly low error is obtained by the Random Forest (RF) algorithm with an average MAE of 37.07 and that for the Gradient Boosting Regressor (GBR) was 36.77. High errors are obtained by the Linear Regression (LR) and Ridge Regression (RR) at 63.53. The Decision Tree Regressor (DT) has a fairly small error of 45.3.

**Fig. 9:**
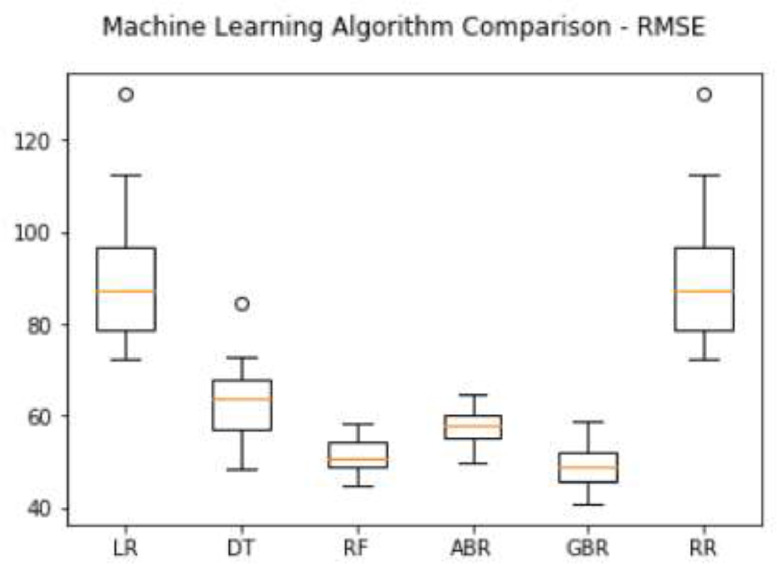
RMSE comparison result.

The measurement results of the 10-fold cross validation are described in the form of boxplots, where one boxplot can represent a general measurement result, the highest measurement results, the lowest measurement results, and the median. As can be seen in Fig. 9, the RMSE measurement results show that a fairly low error is obtained by the Random Forest (RF) algorithm with an average RMSE of 51.2 and that for the gradient Boosting Regressor (GBR) was 49.06. High errors are obtained by the Linear Regression (LR) and Ridge Regression (RR) at 91.9. The Decision Tree Regressor (DT) has a fairly small error of 63.32.

**Fig. 10:**
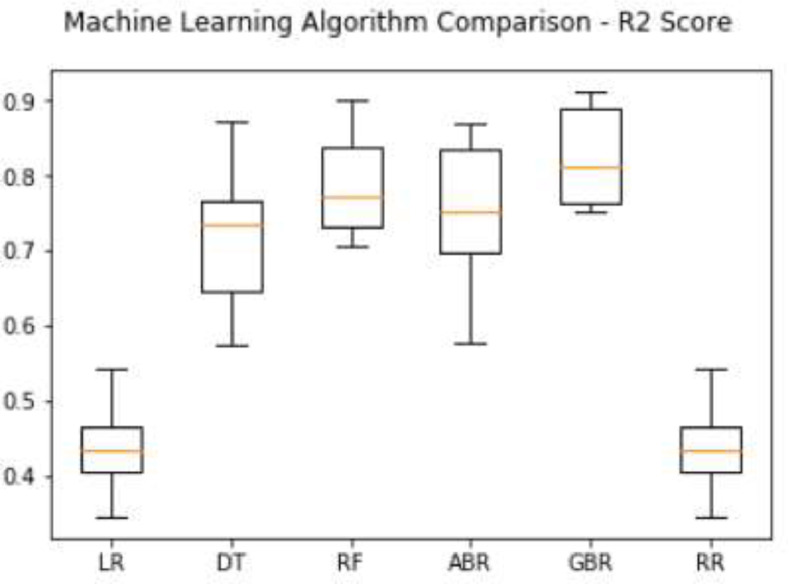
R2 score comparison result.

To assess the quality of the prediction results, we evaluate the R2 score, which is in the range of 0 - 1. The measurement results of the 10-fold cross validation are described in the form of boxplots, where one boxplot can represented a general measurement result, the highest measurement results, the lowest measurement results, and the median. As can be seen in Fig. 10, the measurement results of the R2 Score show that a high enough score is obtained by the Random Forest (RF) algorithm with an average R2 Score of 0.78 and that for the Gradient Boosting Regressor (GBR) was 0.82. Low R2 Score results are obtained by the Linear Regression (LR) and Ridge Regression (RR) at 0.43. The Decree Tree Regressor (DT) has a fairly large R2 of 0.72.

In addition to using the classical machine learning algorithm to model CO_2_, we also use the RNN (Recurrent Neural Network) method. The reason for using the RNN method is because this algorithm is a derivative of deep learning that uses a recurrent network mechanism to handle time series data. It is compatible with the CO_2_ time series dataset. The RNN uses a mechanism to connect the previous state data (t-1) to the next state (t), and so it considers the previous time state (t-1) .

The mechanism of LSTM (Long Short Term Memory) is used in the RNN to model the short term prediction of CO^2^ data. The results we get from the RNN simulation produce an MAE of 39.89 and an RMSE of 56.34. We did not directly compare the results of the RNN method and those of the classical machine learning method because the RNN training was done 50 times and deep learning uses iterative learning for each training step that has been conducted. However, for classical machine learning, training is only done once, and this is enough since it is not iterative learning.

It can be seen from the results of the MAE, RMSE, and R2 Score that the best algorithm that obtains the least error and the highest score is the Gradient Boosting Regression (GBR) with an R2 Score of 0.82, an MAE of 37.07, and an RMSE of 49.06. Therefore, we can conclude that the prediction system using the GBR algorithm is quite good at making short-term predictions of the CO_2_ concentration.

A WSN is a framework that produces huge amounts of information. Hence, a framework that can competently deal with the enormous amounts of data is required. As a solution, we proposed a framework model that can be used as a tool to measure and predict the CO_2_ concentration. The collected data are analyzed by the machine learning algorithm that has achieved excellent accuracy performance. It results in an R2 score of 0.82. In further research, we are going to analyze CO_2_ data combined with another dataset, e.g., CCTV data, to predict the CO_2_ concentration.

## Authors' contributions

• Ari Wibisono: Managed the technical implementation and the testing scenario, and combined all the technical results.
• Hanif Arief Wisesa: Constructed and developed the technical materials of this paper.
• Wisnu Jatmiko: Head of the project, and wrote the research background and introduction
• Ahmad Gamal: Vice head of the project, and wrote the research background and introduction
• Siti Aminah and Aulia Arshad: Conducted the statistical analyses and wrote the results
• Novian Habibie: Integrated and implemented the application and WSN
• Aulia Arshad: Applied, implemented and evaluated the sensor nodes
• Indra Hermawan: Conducted the device configuration and data acquisition
• Aditya Murda: Conducted the WSN implementation and evaluation

## Declaration of Competing Interest

The authors declare that they have no competing interests.
